# Peak oxygen uptake (VO_2peak_) across childhood, adolescence and young adulthood in Barth syndrome: Data from cross-sectional and longitudinal studies

**DOI:** 10.1371/journal.pone.0197776

**Published:** 2018-05-24

**Authors:** William Todd Cade, Kathryn L. Bohnert, Dominic N. Reeds, Linda R. Peterson, Adam J. Bittel, Adil Bashir, Barry J. Byrne, Carolyn L. Taylor

**Affiliations:** 1 Program in Physical Therapy, Washington University School of Medicine, St. Louis, Missouri, United States of America; 2 Department of Medicine, Washington University School of Medicine, St. Louis, Missouri, United States of America; 3 Department of Electrical and Computer Engineering, Auburn University, Auburn, Alabama, United States of America; 4 Department of Pediatrics, University of Florida, Gainesville, Florida, United States of America; 5 Department of Pediatrics, Medical University of South Carolina, Charleston, South Carolina, United States of America; TNO, NETHERLANDS

## Abstract

Barth syndrome (BTHS) is an ultra-rare, X-linked recessive disorder characterized by cardio-skeletal myopathy, exercise intolerance, and growth delay. Oxygen uptake during peak exercise (VO_2peak_) has been shown to be severely limited in individuals with BTHS however; the trajectory of VO_2peak_ from childhood to young adulthood is unknown. The objective of this study was to describe VO_2peak_ from childhood through young adulthood in BTHS. Methods and Materials: VO_2peak_ over time was presented through cross-sectional (n = 33 participants) and a longitudinal analyses (n = 12 participants). Retrospective data were obtained through maximal exercise testing on a cycle ergometer from individuals with BTHS who were or are currently enrolled in a research study during July 2006-September 2017. Participants included in the cross-sectional analysis were divided into 3 groups for analysis: 1) children (n = 13), 2) adolescents (n = 8), and 3) young adults (n = 12). Participants in the longitudinal analysis had at least two exercise tests over a span of 2–9 years. Results: VO_2peak_ relative to body weight (ml/kgBW/min), fat-free mass (FFM) and by percent of predicted VO_2peak_ obtained were not significantly different between children, adolescents and young adults. VO_2peak_ did not longitudinally change over a mean time of ~5 years in late adolescent and young adult participants with repeated tests. A model including both cardiac and skeletal muscle variables best predicted VO_2peak_. Conclusions: In conclusion, VO_2peak_ relative to body weight and fat-free mass demonstrates short- and long-term stability from childhood to young adulthood in BTHS with some variability among individuals.

## Introduction

Barth syndrome (BTHS) is an ultra-rare (1/300,000 births [1]), X-linked recessive disorder characterized by cardio-skeletal myopathy, exercise intolerance, neutropenia, growth delay and 3-methylglutaconic acid [2, 3]. In BTHS, mutations in the tafazzin gene (*TAZ*, located on Xq28), cause abnormal phospholipid metabolism mainly by affecting mitochondrial cardiolipin [4]. Tafazzin, a phospholipid-lysophospholipid transacylase, remodels monolysocardiolipin to tetralinoleic cardiolipin and mutations in tafazzin result in smaller and fragmented mitochondria [5], disruptions in mitochondrial supercomplexes[6], instability in the mitochondrial inner membrane necessary for ATP production [7], and markedly reduced respiratory capacity [5].

Maximum oxygen uptake (VO_2max_), a concept first proposed by Hill et al. in 1923 [8, 9], is defined as the highest rate of oxygen uptake and utilization by the body during intense, maximal exercise that no further increases in work rate bring on additional rises in VO_2_ (i.e. plateau) [10]. Peak VO_2_ (VO_2peak_), directly reflective of VO_2max_, is the highest value of VO_2_ attained upon an incremental or other high-intensity exercise test, designed to bring the subject to the limit of tolerance[11]. VO2_peak_ is a measure that combines cardiovascular and skeletal muscle oxidative function (i.e. Fick equation: oxygen uptake = cardiac output x arterio-venous oxygen difference [12]), as well as pulmonary ventilation and diffusion capacity, and reflects the integrated ability to transport oxygen from atmospheric air to the mitochondria to perform work [13]. VO_2peak_ is reliably measured by indirect calorimetry [14, 15] during graded, maximal exercise, typically performed on a treadmill or cycle ergometer, and is routinely obtained in the clinic and research settings. After adjusting for age and other risk factors, VO_2peak_ is one of the best predictors of cardiovascular [16, 17] and overall mortality [18, 19] in adults and is routinely used in the diagnosis of mitochondrial disease [20, 21].

Our group previously demonstrated severe impairments in VO_2peak_ in individuals with BTHS that was due to a combination of cardiac and skeletal muscle oxygen extraction dysfunction [22]. We also have shown impaired skeletal muscle oxidative function in children, adolescents and young adults with BTHS that was strongly correlated with reductions in VO_2peak_ [23]. Due to the integrative nature of VO_2peak_ in reflecting both cardiac function and skeletal muscle mitochondrial capacity, and the documented cardio-skeletal impairments in individuals of varying ages with BTHS, VO_2peak_ might be an ideal clinical outcome measure for interventional trials in this population. Therefore, establishing the time course of potential changes in VO_2peak_ with age is important in its validation as a clinical outcome measure. However; the time course of VO_2peak_ in BTHS is not known.

Rises in absolute VO_2peak_ (L/min) during childhood through young adulthood in healthy, unaffected individuals corresponds strongly to increases in physical growth[24]. However; VO_2peak_ relative to body (ml/kg/min) weight generally remains stable from late childhood through young adulthood in healthy, unaffected individuals[25, 26]. Although the trajectory of VO_2peak_ across the life span is not known in BTHS, cross-sectional data from the six-minute walk test suggests that cardiorespiratory fitness is higher in children and adolescents compared to young adults and is inversely associated with age in those affected by BTHS[27]. As the six-minute walk test has been shown to be associated with VO_2peak_ in healthy, unaffected individuals[28], these data suggest that VO_2peak_ relative to body weight might decline with advancing age in individuals with BTHS.

The overall objective of the study was to describe VO_2peak_ from childhood through young adulthood individuals with BTHS. Our secondary objective was to examine the relationships between cardiac and skeletal muscle oxidative function and VO_2peak_ in those with BTHS. We hypothesized that VO_2peak_ would be the highest in children and adolescents compared with young adults with BTHS and VO_2peak_ would longitudinally decline over time in participants with serial exercise tests.

## Materials and methods

### Study design

#### Cross-sectional analysis

Retrospective data were obtained from individuals with BTHS who were [22, 23, 29] or are currently enrolled (NCT#011629459) in a research study from July 2006-September 2017. All research was approved by the Human Studies Committees at Washington University in St. Louis or the University of Florida. All child participants provided written assent and adult participants and parents of child participants provided written consent to participation. Participants with BTHS (n = 33) were divided into 3 groups for analysis: 1) children (n = 13, ages 10–15 yrs), 2) adolescents (n = 8, ages 17–21 yrs), and 3) young adults (n = 12, ages 23–32 yrs). Adolescence was defined as ages 17–21 yrs as puberty is typically delayed in BTHS [2] (Table 1). Clinical trials registration: NCT01629459, NCT01625663, NCT01194141.

**Table 1:** Participant demographics, peak exercise testing and echocardiography.

	Children (10–15 yr)(n = 13)	Adolescents (17–21 yr) (n = 8)	Young Adult (23–32 yr) (n = 12)	F-statistic p-value
Age (years)	13 ± 2*,**	18 ± 2**	28 ± 3	0.001
Height (cm)	141.9 ± 12.5*,**	166.7 ± 8.8**	178.4 ± 7.4	0.001
Height z-score	-1.6 ± 1.0	-1.2 ± 1.1		
Weight (kg)	33.9 ± 12.2*,**	55.0 ± 13.9**	67.7 ± 13.6	0.001
Weight z-score	-1.9 ± 1.5	-1.8 ± 1.7		
BMI	16.4 ± 3.2**	19.8 ± 4.5	21.1 ± 3.2	0.007
BMI z-score	-1.3 ± 1.5	-1.6 ± 2.3		
FFM (kg)	29.4 ± 11.9**	37.1 ± 7.7	41.1 ± 6.3	0.02
Fat Mass (kg)	6.7 ± 16.7*,**	22.3 ± 12.1	26.6 ± 11.1	0.001
Exercise Testing				
VO_2peak_ (L/min)	0.5 ± 0.1*,**	0.7 ± 0.2	0.8 ± 0.2	0.008
VO_2peak_ (ml/kgBW/min)	14.6 ± 3.7	13.0 ± 2.9	12.0 ± 3.6	0.20
Predicted VO_2max_ (ml/kgBW/min)	46.1 ± 0.8	44.0 ± 0.5	40.4 ± 1.0	
% Predicted VO_2peak_	32 ± 8	29 ± 7	30 ± 9	0.78
VO_2peak_ (ml/kgFFM/min)	17.0 ± 6.3	18.1 ± 1.3	19.1 ± 4.0	0.57
Peak Work Rate (watts)	40.4 ± 10.7*	57.9 ± 16.2	60.0 ± 11.5^†^	0.001
Peak HR (bpm)	164 ± 22	161 ± 22	155 ± 14	0.53
% Predicted Peak HR	79 ± 11	80 ± 11	81 ± 7	0.91
Peak RER	1.3 ± 0.3*	1.6 ± 0.2	1.5 ± 0.2	0.03
Ventilation (L/min)	27.4 ± 11.8**	39.8 ± 12.4	40.7 ± 10.4	0.01
O_2_ Pulse (ml/beat)	5.4 ± 1.2	4.1 ± 1.8	4.6 ± 1.2	0.21
Resting HR (bpm)	87 ± 11	77 ± 5	78 ± 9	0.07
Resting SBP (mmHg)	101 ± 12	104 ± 9	103 ± 8	0.82
Resting DBP (mmHg)	68 ± 10	69 ± 12	67 ± 9	0.92
Peak SBP (mmHg)	116 ± 17	133 ± 18.7	128 ± 21	0.13
Peak DBP (mmHg)	73 ± 17	77 ± 12	79 ± 10	0.63
Echocardiography				
Ejection Fraction (%)	62 ± 6	52 ± 11	57 ± 13	0.13
Fractional Shortening (%)	38 ± 9	33 ± 3	31 ± 7	0.11
Global Strain (%)	-21 ± 2*,**	-17 ± 1	-15 ± 3	0.001
Skeletal Muscle Oxidative Function				
Tau PCr (s)	69 ± 21	93 ± 30	69 ± 13	0.11
Qmax linear model (mmol/s)	0.54 ± 0.13	0.44 ± 0.15	0.54 ± 0.09	0.32
ATP Oxidative (mM/min)	13.1 ± 4.4	11.6 ± 4.2	11.9 ± 3.5	0.75

Values are means ± SD. BMI: body mass index, FFM: fat free mass, VO_2peak_: volume of oxygen consumption during peak exercise, BW: body weight in kg, RER: respiratory exchange ratio, HR: heart rate, SBP: systolic blood pressure, DBP: diastolic blood pressure, PCr: phosphocreatine, ATP: adenosine triphosphate.

*: different from Adolescents,

**: different from Young Adults, p<0.05.

All exercise tests were conducted on either an upright (12%) or recumbent (88%) cycle ergometer (Lode, The Netherlands) with continuous metabolic measurement (Cardinal Health, Dublin, OH (12%), ParvoMedics, Sandy, UT (88%)) and 12-lead ECG monitoring. For each exercise test, participants cycled at a pedaling rate of 60 revolutions/min. Cycle ergometer resistance for each exercise test began at 10–20 watts and was increased each minute by 5–20 watts until volitional exhaustion. Exercise tests were considered to be maximal if the peak heart rate (HR) was ≥85% of that predicted for age (220 − age) and/or the peak respiratory exchange ratio (RER; VCO_2_/VO_2_) was ≥1.1 [30]. Predicted VO_2max_ was determined as previously described [31]. Body composition (fat-free and fat mass) was measured in n = 28 participants by air-displacement plethymosgraphy (Bod Pod, COSMED, Concord, CA). Resting cardiac function via echocardiography (n = 27) and skeletal muscle oxidative function via 31P-magnetic resonance spectroscopy (n = 23) were measured as previously described [22, 23]. Briefly, reported skeletal muscle oxidative function variables are as follows: 1) Tau phosphocreatine (PCr) is the resynthesis time of PCr following calf muscle exercise, and 2) Qmax linear and 3) ATP oxidative are models that estimate skeletal muscle oxidative capacity based on measured PCr resynthesis[23].

The majority of participants were taking cardiac medications and granulocyte colony-stimulating factor (GCSF) and some were taking nutritional supplements at the time of exercise testing (Table 2).

**Table 2:** Medications of participants.

	n	% of sample
Beta-Blockers	17	52
ACE Inhibitors	14	42
Cardiac Glycosides	11	33
GCSF	9	27
Amino Acids	12	48
Vitamins	9	27
Other Nutritional Supplements	5	15

n = 33. ACE: angiotensin converting enzyme, GCSF: granulocyte colony-stimulating factor.

#### Longitudinal analysis

A longitudinal analysis was performed on two repeated tests in late adolescents and young adults with BTHS (n = 12) who had ≥ 2 exercise tests that were separated by ≥ 1 year (range 2–9 years). In participants with ≥ 3 tests, only the participant’s initial and most recently completed test were compared. No exercise test immediately followed an exercise intervention[29] as to not to artificially affect the results. Nine (n = 9) participants completed two exercise tests, two (n = 2) participants completed three exercise tests and one (n = 1) participant completed four exercise tests.

### Statistics

Cross-sectional Analyses: Normality of the data was analyzed by the Shapiro-Wilk test. A one-way analysis of variance (ANOVA) with Tukey Honesty Significant Difference post-hoc pairwise comparisons was used to compare cross-sectional differences in demographics and exercise variables between children, adolescents and young adults in normal data. A Kruskal-Wallis ANOVA analysis with post-hoc pairwise comparison was used for non-normally distributed data (VO_2peak_ in L/min only). Because this was a retrospective analysis and based on available data, we did not perform an a priori sample size analysis. Univariate relationships between VO_2peak_ and cardiac and skeletal muscle oxidative function in the cross-sectional data were examined by Pearson correlation coefficient analysis. Potential predictors of VO_2peak_ with biological plausibility including cardiac (peak HR, ejection fraction, fractional shortening, global strain), skeletal muscle (Qmax linear, Tau PCr, ATP Ox), and pulmonary function (peak ventilation) variables were entered into a backward step-wise linear regression model.

Longitudinal Analysis: Comparison of repeated exercise tests in n = 12 participants were performed using one-way repeated measures ANOVA. SPSS Statistics software (IBM Corp., Armonk, NY) was used to perform all statistical analyses. Data are presented as mean ± standard deviation (SD) and statistical significance was determined at p<0.05.

## Results

### Cross-sectional analysis

#### Demographics

All presented data were normally distributed except VO_2peak_ expressed absolutely (L/min). As expected, age, height, and weight were different between age groups. Body mass index (BMI) and fat-free mass were greater in young adults vs. children however were not different between children vs. adolescents or adolescents vs. young adults. Children had lower fat mass than adolescents and young adults but fat mass was not different between adolescents and young adults (Table 1).

#### Exercise testing

**Fig 1:**
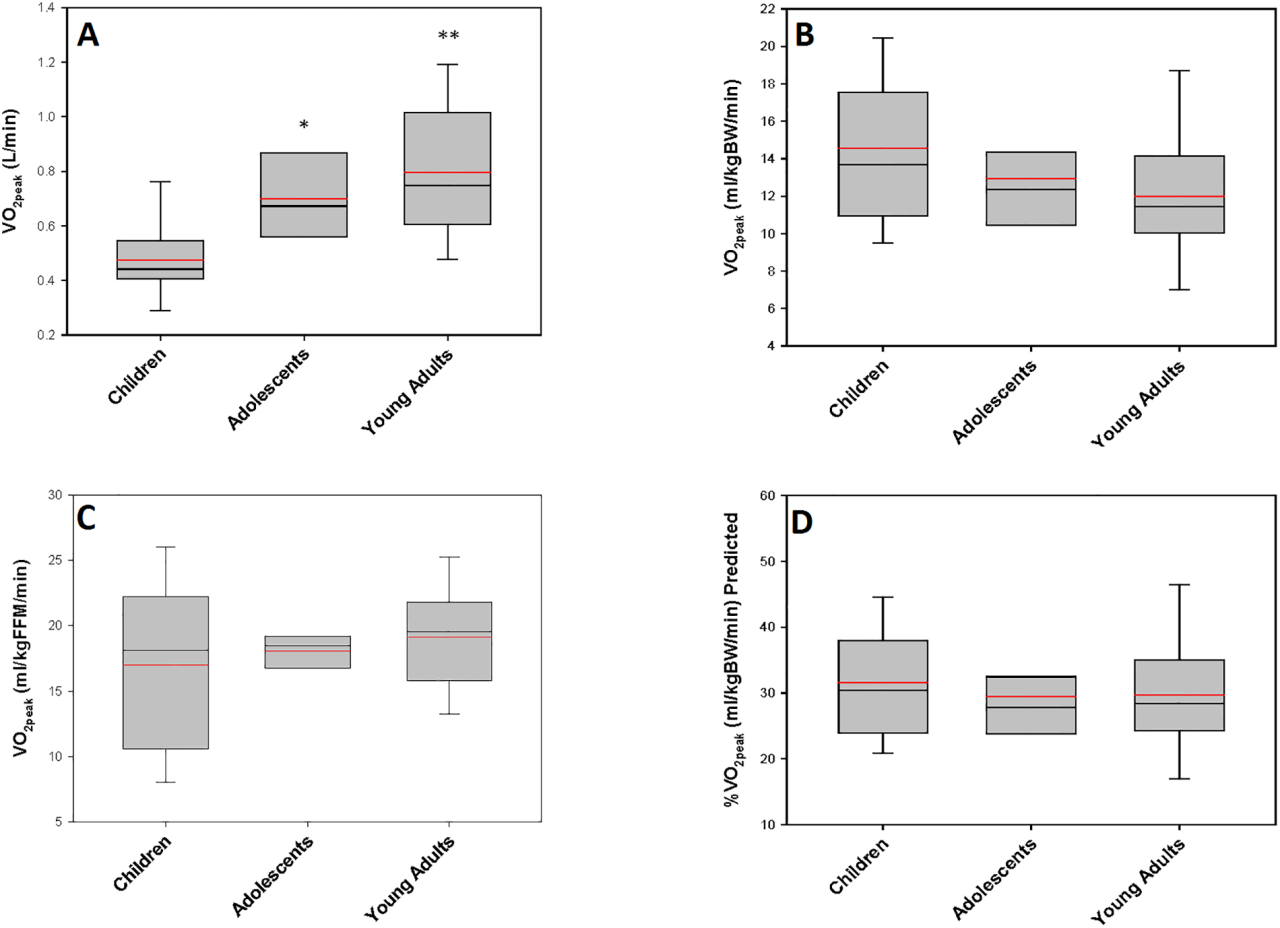
Cross-sectional data on VO_2peak_ in children, adolescents and young adults with BTHS. A. absolute (L/min), B. relative to body weight (kg), C. relative to fat-free mass (kg) and D. as percent predicted for age and body mass [25].

All participants reached an RER ≥ 1.1 and/or ≥ 85% of predicted peak heart rate[30]. Absolute VO_2peak_ (L/min) and peak work rate significantly increased with advancing age group (Fig 1A,Table 1) however VO_2peak_ was not different between groups when expressed by body weight (ml/kg/min) (Fig 1B), fat-free mass (FFM, n = 29) (Fig 1C) or by percent of predicted VO_2max_ obtained (Fig 1D). Peak heart rate (HR), percent of predicted peak HR obtained, peak systolic or diastolic blood pressure, and peak oxygen pulse (VO_2peak_(ml/kg/min)/HR_peak_) were not different between groups. Peak respiratory exchange ratio (RER) was higher in young adults vs. children but was not different from adolescents. Peak ventilation (L/min) tended to be higher in young adults and adolescents vs. children (p = 0.06) but adolescents were not different from young adults. Resting HR or blood pressure were not different between groups (Table 1, S1 File).

#### Cardiac and skeletal muscle function

Resting ejection fraction and fractional shortening were not different between groups however; global strain was significantly lower in adolescents and young adults compared to children. Measures of skeletal muscle oxidative function (phosphocreatine (PCr) recovery time (Tau) and Qmax and ATP oxidative models) were not different between groups (Table 1).

#### Relationships of VO_2peak_ with cardiac and muscle function

Amongst all participants included in the cross-sectional analysis (n = 33) using univariate analysis, VO_2peak_ was associated with peak HR (r = 0.53) and skeletal muscle oxidative capacity (Tau: r = -0.48, p = 0.02, Qmax: r = 0.47, p = 0.03) however was not associated with peak RER, peak work rate, peak ventilation, or resting cardiac function (i.e. ejection fraction, fractional shortening, or strain). VO_2max_ relative to body weight or fat-free mass was also not associated with age (r = -0.30, p = 0.09). A linear regression model including peak HR, ejection fraction and Tau PCr best predicted VO_2peak_ (R^2^ = 0.78, Adjusted R^2^ = 0.73, Collinearity Tolerance: 0.87).

#### Longitudinal analysis

**Fig 2:**
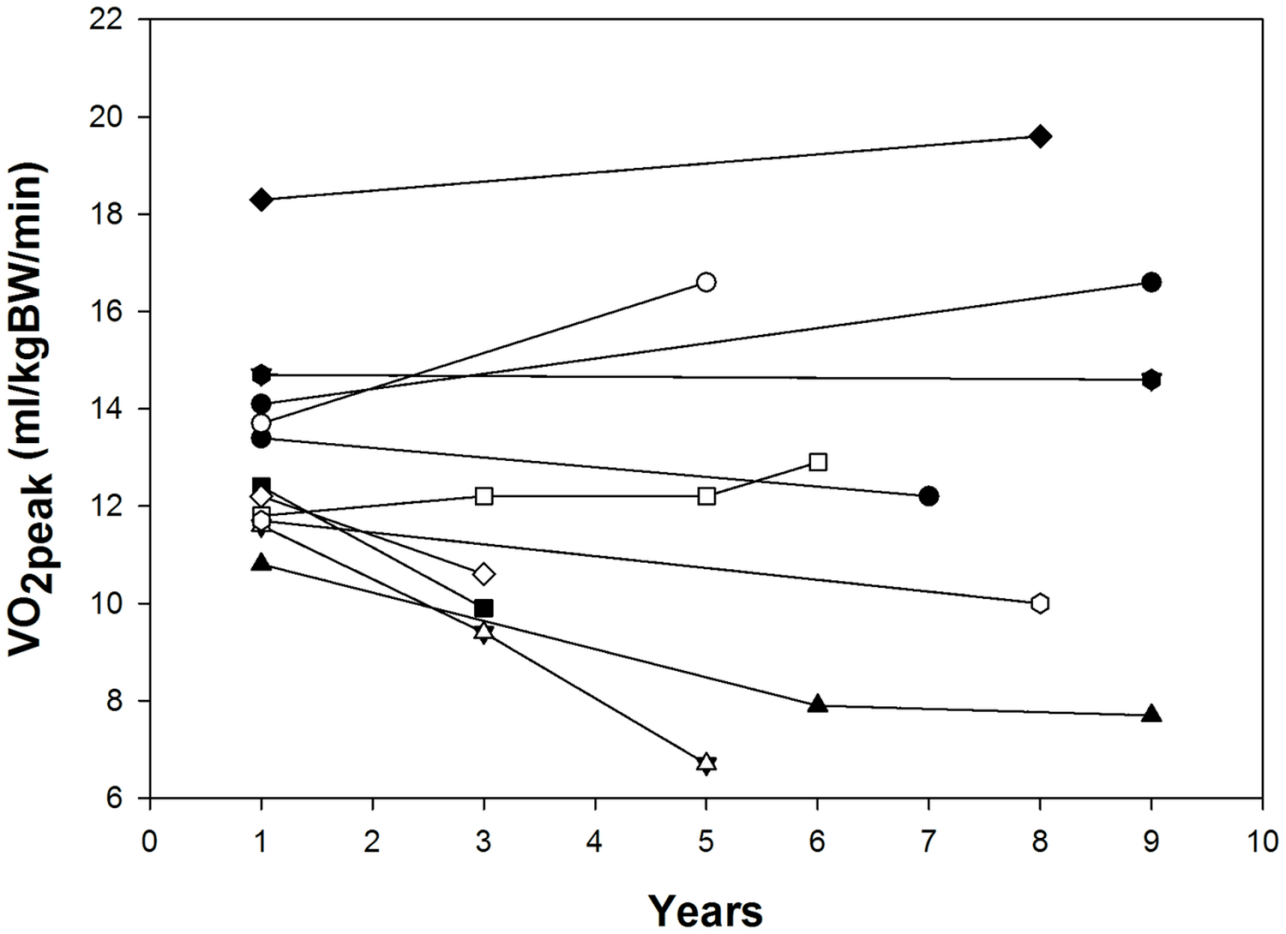
Longitudinal data on VO_2peak_ relative to body mass in individuals with BTHS.

Repeated exercise tests were performed over the span of 2 to 9 years in n = 12 participants (Fig 2). In the analysis of the initial and most recent exercise tests in these participants, weight significantly increased and height tended to increase from initial to most recent test. However, relative and absolute VO_2peak_ and other cardiorespiratory exercise testing variables were not different between repeated tests (Table 3, S1 File).

**Table 3:** Repeater demographics, peak exercise testing and echocardiography.

	Test 1	Test 2	p-value
Age (years)	21 ± 4	26 ± 5	0.001
Height (cm)	161.9 ± 21.2	173.8 ± 12.3	0.07
Weight (kg)	47.9 ± 18.6	57.8 ± 14.0	0.02
Resting HR (bpm)	79 ± 15	80 ± 13	0.94
Resting SBP (mmHg)	98 ± 9	100 ± 6	0.77
Resting DBP (mmHg)	66 ± 5	67 ± 10	0.62
***Exercise Testing***			
VO_2peak_ (L/min)	0.6 ± 0.3	0.8 ± 0.2	0.62
VO_2peak_ (ml/kgBW/min)	12.6 ± 2.7	12.5 ± 3.6	0.91
Peak Work Rate (watts)	52.1 ± 10.4	57.5 ± 11.6	0.63
Peak HR (bpm)	147 ± 18	160 ± 15	0.37
% Predicted Peak HR	73 ± 9	82 ± 7	0.26
Peak RER	1.5 ± 0.3	1.5 ± 0.2	0.82
Ventilation (L/min)	40.3 ± 19.4	45.2 ± 11.5	0.77
Peak SBP (mmHg)	124 ± 22	127 ± 13	0.95
Peak DBP (mmHg)	75 ± 13	78 ± 9	0.56

For participants who had ≥ 2 exercise tests, data presented are the two tests with longest time period between them. Values are means ± SD (n = 14). VO_2peak_: volume of oxygen uptake during peak exercise, BW: body weight in kg, RER: respiratory exchange ratio, HR: heart rate, SBP: systolic blood pressure, DBP: diastolic blood pressure.

## Discussion

This is the first study to describe peak oxygen uptake (VO_2peak_) across the age range in individuals with BTHS. The main and novel finding of the study is that VO_2peak_ (relative to both body weight and fat-free mass) upon graded exercise testing in BTHS appears to be relatively stable as a population from childhood to young adulthood, although some inter-individual variability exists. In addition, VO_2peak_ appears to have short-term stability (~5 years) upon repeated testing in late adolescents and young adults with BTHS. Therefore, due to the short- and relative long-term stability and the integrative nature (i.e. encompassing cardiac, skeletal muscle oxidative function) of the measure, VO_2peak_ may be an ideal clinical outcome measure for intervention studies in children, adolescents and young adults in BTHS.

VO_2peak_ is an integrative measure that combines the processes of cardiovascular, skeletal muscle, pulmonary, and nervous systems to transport oxygen from atmospheric air to the mitochondria to perform physical work [13]. It has been shown to be a strong predictor of numerous health outcomes including cardiovascular and all-cause mortality in healthy, unaffected adults [16–19]. We previously have shown that VO_2peak_ is severely impaired in BTHS that is due to both impaired cardiac function and skeletal muscle oxidative capacity [22, 23]. In the current study, VO_2peak_ in children, adolescents and young adults was approximately 1/3 of the value predicted for age and body mass [31] indicating severe exercise impairment in all age groups of individuals with BTHS. Expressing VO_2peak_ relative to body weight is the most common way of describing VO_2peak_ but expressing VO_2peak_ relative to fat-free mass (although not routinely accessible) is likely a more accurate index as fat-free mass encompasses all non-fat tissue, including heart and skeletal muscle, that is more strongly associated with oxygen uptake than fat tissue[24]. However, similar to VO_2peak_ relative to body weight, we did not find differences between children, adolescents and young adults in VO2_peak_ relative to fat-free mass. Our data contrasts with cross-sectional cardiorespiratory fitness testing (i.e. six-minute walk test) data in children, adolescents and young adults with BTHS. Thompson et al. found lower cardiorespiratory fitness (compared to predicted values) in children and adolescents compared young adults with BTHS and that six minute walk values were inversely associated with age [27]. Even when children and adolescents were combined into one group (n = 21) and compared to young adults (n = 12) in the current study (data not shown), VO_2peak_ relative to body weight or fat-free mass were not different (p = 0.13). The six-minute walk test is associated with VO_2peak_ in healthy individuals [28]; however it is possible that this relationship does not exist in BTHS.

Physical growth is an important contributing factor for the physiologic responses to exercise throughout development [24] and absolute VO_2peak_ (L/min) increases from childhood through young adulthood in healthy, non-affected individuals [32]. This increase in VO_2peak_ with advancing age was seen in participants with BTHS in the current study. Growth in fat-free mass (i.e. skeletal muscle) and heart size (i.e. stroke volume) primarily mediates increases in absolute VO_2peak_ during development [33]. Pubertal status also has a significant effect on VO_2peak_ during development [34]. However, when VO_2peak_ is normalized to body mass, the effects of puberty have been shown to no longer exist indicating that the primary effects of puberty are largely mediated through increases in fat-free mass [34, 35]. This is evidenced by the finding that VO_2peak_ relative to body weight in boys, is highest before puberty and remains stable throughout adolescence [26]. Although VO_2max_ was much lower than predicted for age and body mass [31], the stability of VO_2peak_ relative to body weight was also present in those with BTHS in the current study. Overall, it appears that the trajectory of VO_2peak_ in BTHS, albeit lower, is similar to non-affected healthy individuals.

Based on the findings of the current study, we believe that VO_2peak_, expressed to body weight or fat-free mass, should be considered as a potential clinical outcome measure for intervention trials in BTHS. Based on the definition from the International Council for Harmonisation of Technical Requirements for Pharmaceuticals for Human Use (ICH), a clinical outcome (‘target’ variable, primary endpoint) should be capable of providing the most clinically relevant and convincing evidence related to the primary objective of the trial[36]. In the case of interventional trials in BTHS, it can be argued that cardiac function and alterations in physical function (i.e. exercise intolerance) are the biggest factors influencing quality of life in those with BTHS [37]. Moreover, since resting cardiac function (i.e. ejection fraction) falls within normal limits in many individuals with BTHS[38], a measure (i.e. VO_2peak_) that incorporates both cardiac and skeletal muscle (as well as mitochondrial) function during exercise would be an ideal clinical endpoint in this population. In addition, VO_2peak_ relative to body weight or fat-free mass, does not appear to be greatly influenced by puberty [34, 35]. In the current study we found that a model including both cardiac and skeletal muscle variables best predicted VO_2peak_. This finding, along with the short- and relative long-term stability of VO_2peak_, suggests that VO_2peak_ should be considered as a potential clinical outcome measure for future clinical trials in BTHS.

### Limitations

This study was a retrospective descriptive analysis of a convenience sample of participants complied from studies across 11 years. Due to the retrospective nature of the study, an a priori sample size analysis to determine differences in VO_2peak_ between age groups was not possible and the study might be underpowered to detect differences in VO_2peak_ between age groups. However; the sample size necessary to detect differences in VO_2peak_ between children/adolescents and young adults (i.e. similar to cardiorespiratory fitness differences in the Thompson et al. study[27]), is estimated to be n>100 participants. Age groups assigned in the cross-sectional analysis were grouped upon knowledge of delayed puberty in BTHS[2] however; Tanner staging was not available for all participants so there might have been some overlap in pubertal status between children and adolescents. Most exercise tests were completed on a recumbent (88% for cross-sectional data, 68% for longitudinal data) cycle ergometer and measured by one type of indirect calorimeter (88%, TrueOne, ParvoMedics, Sandy, UT) although there was some variability in exercise mode (upright cycle ergometer) and indirect calorimeter (Cardinal Health, Dublin, OH). However, previously published data demonstrate no differences in physiologic responses to peak exercise testing between upright and recumbent cycle ergometry (including VO_2peak_) in individuals with cardiovascular disease[39, 40]. Although as a population, VO_2peak_ was stable over time, there was some inter-individual variability in VO_2peak_ over time both in the cross-sectional and longitudinal analyses. Worth noting, one individual in the longitudinal analysis had a significant decline in VO_2max_ over 5 years in young adulthood and soon after underwent heart transplantation however; this was unusual as most individuals with BTHS who have to undergo heart transplantation are infants and toddlers [41]. Lastly, echocardiographic, magnetic resonance spectroscopy and body composition data were not available for all participants.

## Conclusions

In conclusion, VO_2peak_ relative to body weight and fat-free mass demonstrates short- and long-term stability from childhood to young adulthood in BTHS with some variability among individuals. VO_2peak_ appears to be a reasonable clinical outcome measure in future intervention trials in BTHS.

## Supporting information

S1 File Cross-sectional and repeater VO2 data in participants with Barth syndrome. (XLSX)
